# Resilient cultural practices for cognitive development during childhood within learning pathways with support from mediated mutual reciprocal theory

**DOI:** 10.3389/fpsyg.2022.994156

**Published:** 2023-03-27

**Authors:** Therese Mungah Shalo Tchombe

**Affiliations:** Department of Educational Psychology, Faculty of Education, University of Buea, Buea, Cameroon

**Keywords:** resilience, cultural practices, learning pathways, cognitive development, mediation, mutuality, reciprocity

## Abstract

**Introduction:**

This paper examines resilient cultural practices, informed by cultural values for cognitive development in socialisation during childhood within the learning pathways model. It argues that the active role of children in their learning is not well captured or explained by theories or even empirical data. Learning pathways as a model in this paper is significant because it orients thinking on the fact that all activities children engage in always have social, psychological, and physical implications for cognitive enrichment. The activities are driven by resilient cultural practices informed by cultural norms, beliefs, and values such as responsible leadership qualities and cultivating socio-emotional and moral balance. With the above reasoning, this paper is underpinned by an empirical study with six specific objectives supported by Mediated Mutual Reciprocity theory.

**Methods:**

The study used a mixed research design and conveniently selected a sample of 73 parents and teachers whose ages ranged from 25-50 years. A questionnaire and an interview guide were used for data collection. The three-sections questionnaire was constructed to find out information about resilient cultural practices and cognitive development. The items in sections two and three were rated on a 5 points Likert scale based on the occurrence of practices and behaviours. The interview guide was constructed to find out information on the three learning pathways; physical, social and psychological that are relevant to children in African cultures. Data were analysed using frequency and proportions and Multiple Regression Analysis to aggregate scores for given conceptual components. Analysis of qualitative data followed the systematic process of thematic and content analysis.

**Results:**

Based on qualitative findings, it was realised that African children are exposed to physical, social, and psychological pathways to learning. Quantitative results showed that 85.5% of respondents are high in their resilient cultural practices whereas 14.5% of them are low in resilient cultural practices. In the same line, 74.4% of respondents’ cognitive development is high while a proportion of 25.6% is low. The inferential statistics showed that resilient cultural practices are significantly predictive of cognitive processes, reasoning, skills, and strategies at a 0.000 level of significance.

**Discussion:**

Based on the use of the principles of the Mediated Mutual Reciprocal Theory, the study affirms the importance of children’s contributory role in their learning and cognitive development. The link between pathways, resilient cultural practices, and cognitive development highlights the significance of children’s involvement in their development through participation.

## Introduction

African traditional perception of learning refers to a socialisation process by which children acquire knowledge through psychological, social, and physical pathways peculiar to African cultures (Merriam, 2007). The Africentric learning pathways through various cultural activities generate subject matter specific to physical, social, and psychological aspects in development. The subject matter addressed by each pathway does not exist in isolation. The holistic view dominating development in African cultures prevails. Thus, the pathways are interwoven. Addressing each independently is for clarity and understanding of the functions of the pathways.

The learning pathway is the chosen route taken by children/learners through a range of psychological, social, and physical activities, which allows them to build knowledge progressively and acquire cognitive skills. With the rise of globalisation, society imposes learning pathways upon African children that do not meet their needs or fit their expectations. The relationship between a people’s culture and learning pathways for cognitive enrichment is critical because learning is contextual to the physical, psychological, and social environment in which children grow and develop. What most scholars consider as learning is limited to Eurocentric epistemologies, methodologies, and theories (Nsamenang, 1992). These are alien to African perspectives as these do not address the real contextual and developmental needs of African learners. Rogoff (2003) argues that we need to consider and understand that cognitive development is a cultural process. Individuals develop as participants in their cultural communities, engaging with others in shared endeavours and building on cultural practices of prior generations (Nsamenang, 1992, 2008). If the learning process is to be functionally relevant for the African peoples, it should maintain the dynamic patterns of continuity with the family and the cultural life of the people (Onwauch, 1972).

Resilience, or the processes of positive adaptation to significantly difficult life circumstances, is influenced by the culture and context in which an individual is embedded (Masten et al., 2010; Ungar, 2011). Increasingly, researchers have addressed how Africentric cultural aspects that influence resilience. While a general description of the values, beliefs, and of people of African descent, or an Africentric way of being, has been questioned by some. Schiele (2000), among others, defended this generalisation, given that ‘conspicuous commonalities existed in core philosophical assumptions across the various ethnic groups. These assumptions fundamentally validated an ontological and cosmological framework that underscored a collective and spiritual worldview’ (p. 17). Various other scholars have offered quantitative empirical proof that an Africentric worldview encourages practices and beliefs that nurture African Americans’ resilience (Jones, 2007; Utsey et al., 2007; Brown, 2008; Neblett et al., 2010). Their studies have foregrounded social support networks (including formal and informal kin), collective coping, and spirituality/spiritual coping as cultural mechanisms that mediate adversity and encourage resilience. This section examines three aspects of African cultural practices that tend to promote resilience.

Resilient cultural practices employed during socialisation build children’s competencies and skills which support their actions through learning for quality development (Yatta, 2007). The learning pathways adopted for this paper inform on the alleyways through which learning takes place such as physical, psychological, and social spaces, in which children dynamically create and acquire knowledge. This paper argues that learning pathways are under the control of children because the activities are needs and interest-driven even if caregivers, teachers, or parents create enabling environment (Piaget, 1952; Tchombe, 2011, 2014, 2019). Adopting the physical, social, and psychological learning pathways, the paper establishes another dimension for viewing learning and development as holistic, a further perspective of the Africentric worldview. This is because it is not evident that Euro-American theories have the potential to fully explain the active involvement or the actions/processes or the outcomes that are considered determinants of a socially competent person depicting an intelligent child from an African worldview. African cultural practices in socialisation have always addressed skills for survival, higher-order cognitive learning, and other competencies required for the 21^st^ century. African children are engaged in diverse activities and actions stretching the use of their cognition, social even moral, and emotional skills early in life. These reflect cultural beliefs and values informing activities for promoting skills and competencies in overcoming life challenges in growing up.

Learning is contextual, situational, and informed by cultural values. Rogoff (2003) argues that we need to understand that cognitive development is a cultural process. Individuals develop as participants in their cultural communities, engaging with others in shared endeavours and building on cultural practices. Even, if the learning process is to be functionally relevant for Africans, it should maintain the dynamic patterns of continuity (Onwauch, 1972). The relationship between a people’s culture and learning pathways for cognitive enrichment is critical because learning is contextual to the physical, psychological, and social environments in which children grow and develop (Merriam, 2007). Through the social pathway, they cultivate social relationships, survival skills, leadership skills, and responsible. The psychological pathway enables the use of executive processing abilities, and skills using quality cognitive strategies. Physically, through manipulating their immediate environment, they come to terms with its realities through all forms of collaborative hands-on activities, play, and other cultural engagements as exemplified in the pictures below.

What still constitutes learning in Africa rather draws from Eurocentric epistemologies, methodologies, and theories. Euro-American theories lack insights into African ability to acquire information, develop positive relationships and demonstrate self-regulating emotional skills. Hence, this paper relies on the Mediated Mutual Reciprocal (MMR) theory to explain how African children have a central and active position in learning (Tchombe, 2019). What the MMR theory tries to propose among others is the interactive and interconnected roles of learners, competent others, and the environment. The assumption, therefore, is that the process is not divisible but a chain of cyclical actions for appropriate interactions and eventual positive quality outcomes. Like Feuerstein’s (1990) Mediated Learning (ML), the MMR ascribes initiated autonomous functioning for the learner through mediated mutual reciprocal action. The theory focuses on explaining the learner’s actual contributions in the construction of knowledge such as what he/she does in concrete terms. Learners easily assume responsibility and ownership of the whole learning process by changing the frame of reference through their own engagements in the MMR action by reacting, negotiating, questioning, and providing alternative views as they engage in all the above tasks (Tchombe, 2019). Göbel et al. (2013) argue that reciprocity is a form of interaction that essentially centers on mutuality. Tchombe (2014, 2019) adds that children’s mediation is an essential aspect of this exchange. Children’s active involvement in such interactions needs to be well researched and supported by theories sensitive to Africentric epistemologies.

This paper, therefore, examines resilient cultural practices for cognitive development and learning pathways. The paper highlights a recent empirical study conducted by Tchombe (2021) on resilient cultural practices for cognitive development during childhood within learning pathways.

## Mediated mutual reciprocal theory and the understanding of Africentric pathways to learning

The Mediated Mutual Reciprocity (MMR) theory can enhance understanding of learning pathways as the theory highlights another dimension of socio-cultural constructivism with more accents on the learner’s inputs through their initiated interactions and actions. The learning approach encouraged by MMR is co-learning and non-hierarchical based on worthwhile activities that are interdependent and socio-culturally relevant (Tchombe, 2019). The learning process gearing towards transformative learning is deep structured requiring more, the total engagement of the learners. This theory is purposeful, useful, and meaningful in helping to explain children’s and learners’ own contributory role in directing their learning and development. Very early, children mediate and mutually reciprocate in the process of knowledge creation, utility, evaluation, and dissemination. Mediated Mutual Reciprocity is a give-and-take process. The teachers or any competent others, including the learners/children, give and equally receive; thus, affecting and influencing each other’s behaviour, sometimes positively redirecting, and changing the course of the learning process. The theory prescribes the great need to understand learners’ input actions as their mediated mutual reciprocity is need-interest-driven (Tchombe, 2006, 2011). This intrinsic motivation promotes the use of different technological and communication tools and styles given their status through diverse actions. It informs that the locus of control is more internal to the learner not so much external. Mediated Mutual Reciprocity is not so much generating knowledge but transforming the knowledge into ideas that can be used to create or redirect actions, requiring more learner involvement. Learners’ communicative behaviours in these contexts illustrate they can influence and be influenced by the behaviours of others. The dialectical relationship generated, illustrates that all the actors influence each other’s behaviours in significant ways as they recognise, negotiate and exchange ideas in the transaction through a cyclical chain, thereby causing bidirectional changes. The process illustrates industry, reflection, reasoning, and engagements. The mediating effects of the rich empirical world of the African cultural context provide opportunities for the discovery of specific knowledge, skills, competencies, attitudes, and values that no one other than the learner can discover and exploit through an independent mutual reciprocation process All of these actions are also encouraged by cultural amplifiers such as farming fishing, hunting marketing, and traditional activities. It is hoped that the Mediated Mutual Reciprocity perspective will permeate home and school attitudes to sustain children’s and learners’ own inputs in the development of their creative thinking, problem-solving, analytic, and self-reinforcing skills, and abilities. The theories underscore the active nature of African children in learning for cognitive enhancement It also illustrates how Africentric learning contexts enable children to initiate/sustain a quality learning process; valuable in ensuring learners’ own significant contribution to learning that is dynamic and sustainable (Tchombe, 2019).

### Difference between MMR and Vygotsky in the understanding of learning pathways

The increasing shift from teaching to learning (Tchombe, 2006) provides a new educational perspective in which the learner is required to assume an active rather than passive role in the construction of knowledge in formal education in Africa. Social constructivists also address situations of learning, where the learner takes part in activities that are directly relevant to the application of learning and which takes place within a culture like the applied setting (Brown et al., 1989). The underlying principle of Vygotsky (1978), social constructivism is clear, on the fact that the learner’s own reality is shaped by his/her background, culture, or embedded worldview. Vygotsky (1978) suggests that knowledge is constructed through mutual interpretations of the world through cooperative and collaborative learning in ways that would not be possible if learners functioned autonomously (Greena et al., 1996). Interactive learning includes reciprocal teaching, peer collaboration, and cognitive apprenticeship (Vygotsky, 1978). Through a process of ‘scaffolding’ such as questioning for example the capacity of the learner can be extended beyond the limitations of physical maturation to the extent that the development process lags behind the learning process (Vygotsky, 1978). Children’s backgrounds and cultures are essential mechanisms influencing the learning process (Wertsch, 1981).

From the above reflections, it is obvious that Vygotsky to an extent failed to fully address the experiences of African children. It is time we reflect on the input capacity of learners and the roles of competent others including the contexts, tools, and tasks during formal and informal or non-formal learning. The development of children’s knowledge, skills, competencies, and attitudes for purpose of enhancing and enriching cognitive skills and processes are not shaped only by competent others. The significant contributions also of the learners must be explained to enable understanding of the extent to which quality learning can be determined by the quality of learners’ engagements. Unfortunately, the focus on inputs in any learning situation for knowledge advancement is more on the contributions of the competent others, not necessarily the learners. So, the actions undertaken in mediating are directional. The teachers or competent others direct learning behaviours through different forms of interactive behaviours such as questioning, describing, explaining, prompting, probing, initiating, and negotiating. What is expected of the learner most often is the mechanistic role of responding. The argument that this pose is that competent others mediated actions, set out most often to achieve directed goals at the Zone of Proximal Development (ZPD; Vygotsky, 1978). Such actions do not explain the learner’s own mediated actions to negotiate change or provide new reactions to the behaviours of competent others that are attuned to the learner’s own interests and learning needs. It is the search for explanations about the part played by the learner that has motivated the shift in focus from directional to multi-directional processes in learning. In a multi-directional action, the learner is challenged more to reflect, analyse, synthesis, evaluate, imagine, create, and solve problems not only of cognitive nature but interpersonal in negotiation and resolving conflicts.

Accordingly, when learning is single-directional from the teacher (significant other) to the learner, the learner remains a passive receptor of knowledge. But in a bidirectional and multidirectional relationship, the learner is an active and co-constructor of knowledge in a non-hierarchical relationship. This raises the question of whether the mediation process can be shifted from competent other’s monopoly or domination to including the child/learner and other mechanisms. To provide an answer to this question, the child/learner’s mutual reciprocal act is seen as the process by which learners engage in the mediation process. This critical action of the learner in the learning/teaching game, if well explained based on this model should illustrate the depth of the learner’s active engagement. This can be demonstrated when the child/learner is seen to mediate on his/her own terms and affect a change in the behaviour of the competent others. On this premise, mediation through mutual reciprocity is central to learning that is transformative. This principle is significant in the mediated mutual reciprocity theory as explained below:

- Mediation: The child/learner and adult are both co-constructors of knowledge. But the actual making meaning and transforming process is done by the learner through internal processes.
- Mutuality: Active interdependence and collaboration between the child/learner and the adult with much input in the transaction from learners transformed ideas strengthening his/her capacity for sustained learning.
- Reciprocity: A learning stimulus initiates a shared responsive connection between the child/learner and the adult enhancing the utility value of the transformed ideas from the co-constructed knowledge

## Africentric pathways to learning

Within African cultures, the harmonious relationship between the different spheres of existence blurs the distinction between the subject and object of knowledge (Tape, 1993). Thus, the African ontology sees humankind as part of nature (Dasen, 2011) which guides the methodology by which the study of nature is supposed to be approached in Africa. This line of thought permits the integration of diverse ethnocultural realities and disparate theoretical threads into a common conceptual system. The embedded knowledge, skills, and values are massed together as integral to physical, social, and psychological interactions (Nsamenang, 2005). The communication patterns of these cultures are conceptually associated with the cognitive abilities of concrete experience, leaving from observed realities to general theories (Inductive approach). In this regard, the Mediated Mutual Reciprocity theory being proposed as a new framework for understanding learning for cognitive enhancement highlights another dimension of socio-cultural constructivism with more accents on the learner’s inputs through their initiated interactions and actions. Before engaging in a discussion of this theoretical approach, let us first consider the various pathways to learning.

### Physical pathways to learning

The physical pathway to learning is meant to assist learners to adjust and adapt to the natural environment to exploit and derive benefit from it in a harmonious relationship (Mundy-Castle, 1974). Gardner’s theory of multiple intelligences illustrates the learning pathways. As one of the components of cognitive development, Gardner considers naturalistic intelligence as the ability to identify patterns in nature and to determine how individual objects or beings fit into them (Gardner, 1999). Within the African context, the child can know how to make use of the natural environment around him or her as a physical pathway to learning. This is the contextual and practical aspect of intelligence and reflects how the child relates to the external world about him or her. Intelligence is an adaptation to, shaping of, and selection of real-world environments relevant to one’s life (Sternberg, 1985). Nature intelligence is highly valued amongst cultures of Africa. In this regard, a child growing up in a typical African indigenous context is required to be able to; identify medicinal plants, do basic plant concoctions that cure basic illnesses, produce play objects and house furniture like chairs and brooms out of natural (bamboos) and waste material, identify cultural symbols and objects like trees, rivers, insects, plants, animals that are of spiritual value and even identify poisonous plants (Sternberg et al., 2001; Yatta, 2007; Nyota and Mapara, 2008).

### Social pathways to learning

The social pathway to learning is dominated by a communal lifestyle and activities whereby living, working, and playing together, feeling for one another, sharing and, collective judgments are key elements of the social order. Communal lifestyle, with key elements of the social order, are respect for elders and social hierarchy, sustenance of good friendships, appreciation of social obligations and responsibilities, conflict management, caution towards strangers, and subordinate individual interests to those of the wider community (Tiberondwa, 1978). Nsamenang (2005), reminds us that the family is the primary agent of socialisation and the pathways to learning make the deepest impression on the child’s intellectual development because it provides the first cultural experiences and training in cultural learning. Elam (1968) states that the African conception of a family consists of households related to each other. Nyota and Mapara (2008) highlight two important activities in the social pathway to learning: traditional games and play, and songs with peers and siblings. According to Gyekye (1995), stories (folktales) and proverbs are primary ways through which a great deal of African philosophical thoughts, knowledge, and wisdom have been taught provoking a lot of reflections. Through the social pathway, children learn to interact with others around them. In doing this, they develop and perceive their own individuality thus gaining skills on how to communicate and process their actions in communal life. Contents in the social pathways provide opportunities through which children/learners engaged in activities enhancing cognitive development (Bidzan-Bluma and Lipowska, 2018).

Thus, through parents, the family lays the foundation for learning for later life. Children learn from their parents and siblings through three principal ways: observation, imitation, and co-participation (Nsamenang, 2005). Children accompany parents to the farms and through this, they learn how to perform their agricultural and farm duties (Rende, 2014). Within the African context, a male child will learn activities like hunting and clearing through interaction with the father, while the female child learns household activities like cooking through interaction with the mother (Yatta, 2007).

Outside the household, the child interacts with the extended family and society at large. Berger (1999) has remarked that if a child’s learning is not aroused by his or her parents, it may be aroused and powerfully when the child begins to compare his or her skills with those of other children of the same age through interactions in the pathways. In the same vein, one of the activities of the social pathway to learning that is used by parents, older siblings, and elders to socialise the African child to acquire cognitive skills is folklore (folktales and proverbs). According to Gyekye (1995), stories (folktales) and proverbs are primary ways through which a great deal of African philosophical thoughts, knowledge, and wisdom have been taught. The oral tradition found expression in stories, folktales, anecdotes, proverbs, and parables. These provoked a great deal of reflection. African knowledge, myths, philosophies, liturgies, songs, and sayings have been handed down by word of mouth from generation to generation. Through these oral media, children are educated by parents and the community on people’s conduct and moral values.

Worthy to note is the fact that proverbs and folktales throw light on the concrete reality of lived experiences and serve as important pedagogical devices because they provide experiential case material on which pedagogical reflection is possible (Manen, 1990). Proverbs and folktales can be considered a subset of the social learning pathway engaging, the political, ethical, and religious ideals of the people among whom they originate. In the words of Olatunji (1984), proverbs serve as a social element with the character to praise what society considers to be virtues such as tolerance, responsibility, dedication, love, discipline, justice, etc. In the same way, proverbs are used to condemn what society considered injustice, intolerance, destruction, jealousy, envy, hatred, and sexual immorality among others (Ajibola, 1947).

### Psychological pathways to learning

Khootorskoy (2005) considers the psychological learning pathway as the way to develop each child’s/student’s educational potential, which is the set of his/her intellectual, creative, and other innate abilities. Scholars see individual learning pathways as a certain sequence of single learning activity components, developed for each child according to their own learning goals, their innate abilities, personal interests, and motives.

The psychological pathway to learning is viewed by Tchombe (2011) as an interest-driven strategy that stems from the parental belief that children must actively be part of all family activities. The importance is not only that children are actively engaged in these activities but that the activities are of interest to the children. Children are made to see the relevance, meaningfulness, and value of the activities in which they engage as they collaborate and generate new knowledge through the participative process (Tchombe, 2011). The interest-driven strategy implies that children are motivated intrinsically to be involved in whatever learning that the activities encourage because of the usefulness of the acquired knowledge. The underlying principle in the interest-driven strategy is learning that applies to the child’s everyday life as s/he relates to others, enabling him/her to be able to understand and provide solutions to life problems. It is not only indicating a significant role for the child in learning but that s/he is the focus of that learning process. In this case, the child-centered principles prevail (Tchombe, 2011).

The psychological pathway to learning, therefore, takes cognisance of the fact that each child perceives (understands and explains) the world differently from other children based on background knowledge, which is idiosyncratic and learning style which is personal. Background knowledge is a particularly important concept in the framework of cognitive theory as it defines the world knowledge that the student has already acquired. The new experience is usually compared to the experiences already stored in the memory which helps to better understand what is going on. The role of previous knowledge is valuable as it informs on children’s existing capacity. Any information either spoken or written does not by itself carry meaning. Rather, it only provides directions for children/students which help construct meaning from their own, previously acquired knowledge. We can say that comprehension is an interactive process between the student’s background knowledge and the information to be learned. Efficient assimilation requires the ability to relate the information to one’s own knowledge.

## Resilient cultural practices for cognitive development

Resilience, or the processes of positive adaptation to significantly difficult life circumstances, is influenced by the culture and context in which an individual is embedded (Masten et al., 2010; Ungar, 2011). Resilient cultural practices directing socialisation see all activities involving children as holistic learning opportunities in which children acquire knowledge, competencies, and values. These inform children’s capacity how to respond to challenges. It harnesses children’s processing capacity to adapt to challenges and the pathways provide opportunities to use patterns of adaptive functions reflecting resilience (Masten et al., 1990). Such activities address the psychological, social, and physical components of the pathways to development. Such pathways according to Africentric views are unique to African cultural socialisation patterns and are thus (unlike Euro-American) culturally specific (Merriam, 2007). Resilient cultural practices, therefore, build children’s capacity through multiple processes based on cultural beliefs and actions to help children develop appropriately against all odds. Resilient cultural practices address activities specific to holistic development. The subject matter addressed by each practice does not exist in isolation. Resilient cultural practices could include personal survival skill practices, leadership practices, a sense of responsibility, and the development of socio-emotional and moral value practices. Developing these competencies through such practices, makes the children socially competent persons, responsible in their respective communities.

### Development of leadership and responsibility skills through symbolic, handovers observations and guided participation in daily routines

**Figure 1:**
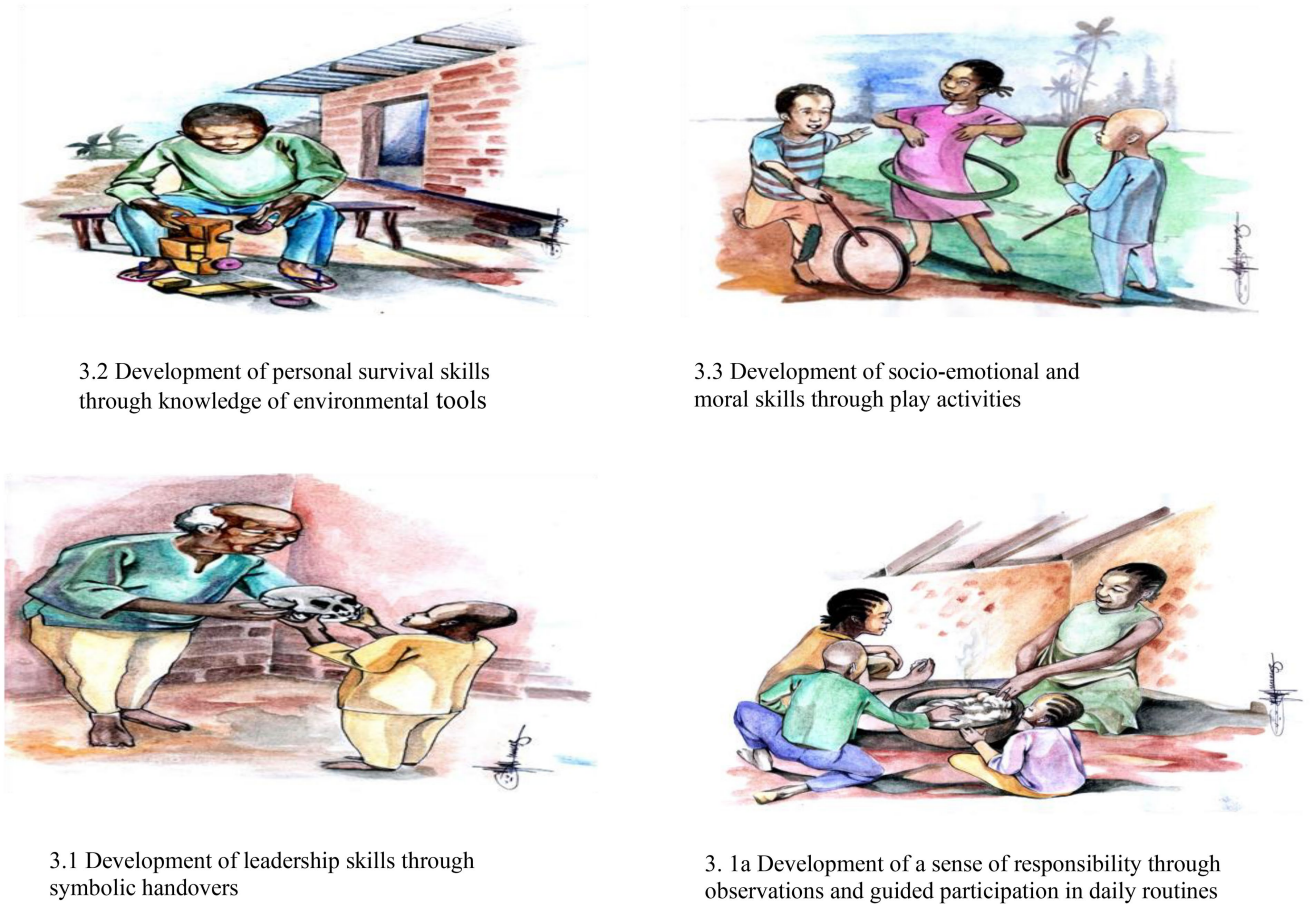
Resilient cultural practices for cognitive development. Source: Pictures adapted and drawn from current write-up (Noumsi, 2021, unpublished).^1^

With the possession of symbols such as tools and language, the child embraces leadership and begins to learn how to cultivate leadership skills. The symbols remain a source of strength and continuity. Such a symbol could be handed over from generation to generation within the family line of succession (Yatta, 2007) because the basis of its development is through relevant cultural values and practices. A child’s participation in family life through the performance of daily routines is of capital importance to African parents and elders (see Figure 1). Household chores include the child’s ability to perform activities such as cleaning duties, running errands, fetching objects like wood and water for the family, and cooking (Rende, 2014). Children are socialised into these intelligent behaviours through observation and imitation of parents and older siblings (Yatta, 2007).

### Development of personal survival skills through environmental adaptation

Nature intelligence is highly valued amongst cultures of Africa. In this regard, a child growing up in a typical African indigenous context is required to be able to; identify medicinal plants, do basic plant concoctions that cure basic illnesses (See Figure 1), produce play objects and house furniture like chairs and brooms out of natural (bamboos; see also Figure 1) and waste material, identify cultural symbols and objects like trees, rivers, insects, plants, animals that are of spiritual value and even identify poisonous plants (Sternberg et al., 2001; Yatta, 2007; Nyota and Mapara, 2008).

### Development of socio-emotional and moral skills through play activities

Socio-emotional and moral skills, constitute the child’s ability to make and sustain healthy relationships with peers and other members of the community through the exhibition of pro-social intelligent behavior. Within the African context, Nyota and Mapara (2008) reveal that through games and plays, and singing with peers, children socialise themselves into acceptable interpersonal relationships. These include the child’s ability to; give and receive help from peers, and keep friends and playmates (see Figure 1). They also manage conflict, learn future gender roles, manage success and failure, live and work together with others, and participate in community tasks. Accordingly, they celebrate with others and feel for others in times of worry and distress.

## Empirical study on resilient cultural practices for cognitive development during childhood within learning pathways

Section two of the paper highlights a recent empirical study that addresses the conceptual and theoretical considerations of the paper. This study was conducted by Tchombe (2021) on resilient cultural practices for cognitive development during childhood within learning pathways. The section highlights the specific objectives of the study, materials and methods, findings, discussions, conclusion, and recommendations.

### Objectives of the study

The general objective of the study was to examine the effects of resilient cultural practices on cognitive development and to identify the learning pathways that are relevant to children in African cultures.

The specific objectives of the study were:

- To examine the effects of leadership/responsibility on cognitive development.
- To determine the effects of personal survival assets on cognitive development.
- To examine the effects of socio-emotional values on cognitive development.
- To determine the effects of socio-moral values on cognitive development.
- To identify learning pathways that are relevant to children in African cultures.

### Materials and methods

#### Design

A mixed research method was adopted for the study, using a triangulation approach where the questionnaire constructed had both closed and opened ended questions for data collection. The qualitative questions were to provide data to expand quantitative results on the various components of resilient cultural practices on cognitive development. However, an interview guide was designed to identify the various learning pathways with support from Mediated Mutual Reciprocity theory.

#### Sampling and ethical procedures

The target population for this study included adults of ages 25 to 50. These formed the respondents that were required to achieve the five objectives of the study. Due to the outbreak of COVID-19 pandemic, a convenient sampling technique was used for the study based on respondents who were identified and were able to complete the online google form. In this regard a Google form was created, a link that could be shared *via* email or social media to get answers from respondents. A total number of 90 questionnaires were given out to respondents.

Respondents were recruited through a phone call and procedures of completing the online form were explained to them as well as the ethical considerations. The ethical issues related to this study focused on the research participants and the author. As far as the participants are concerned, the ethical issues to address to ensure the quality and validity of the data and the study at large consisted of explaining to the participants the significance and helpfulness of the study and justifying why it was important for them to participate in the study. In line with this, the author made sure the participants were adequately aware of the type of information needed from them, why the information was being sought, what purpose it was to be put into, how they were expected to participate in the study, and how it directly or indirectly affected them without pressurising them to consent. On the part of the author, ethical considerations consisted in avoiding bias, using appropriate research methods; using appropriate methods to validate the data collection instruments, and doing correct reporting of findings without falsifying them.

**Figure 2:**
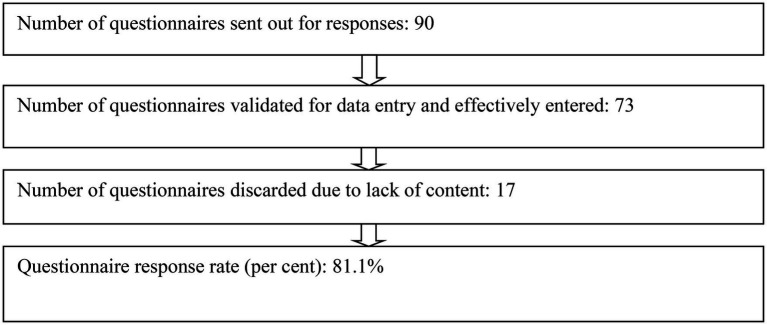
Questionnaire response rate.

Respondents were informed that those not interested are free to withdraw at any time from the study without any sanction and finally that the anonymity and confidentiality of their responses will be ensured. When participants gave their consent to the study, they were then sent the online form link *via* their emails or WhatsApp numbers based on their preference. A period of 2 weeks was given for respondents to complete the questionnaire. The progress of responses collected was monitored by all those added as collaborators. A response of 73 respondents was realised at the end of the data collection period as depicted in Figure 2.

Figure 2 shows that 90 questionnaires were sent out to respondents, 73 were validated for data entry and effectively entered. 17 were discarded due to a lack of enough information and content for analysis. This gave a response rate of 81.1%, which was deemed satisfactory for the study.

The respondents were made of diverse groups in relation to age, gender, and place of residence as seen in Table 1.

**Table 1:** Demographic information of respondents.

Demographic information		Frequency	Percentage
Age	Less than 30	10	13.7%
	30–40	31	42.5%
	41–50	21	28.8%
	51+	11	15.1%
Gender	Female	39	53.4%
	Male	34	46.6%
Place of residence	Rural area	3	4.1%
	Urban area	70	95.9%

Table 1 showed that out of the 73 respondents, 10 (13.7%) are less than 30 years, 31 (42.5%) are 30–40 years of age, 21 (28.8%) are 41–50 years and 11 (15.1%) are above 50 years. Gender-wise, 39 (53.4%) are female and 34 (46.6%) are male. Finally, based on place of residence, most respondents 70 (95.9%) stay in an urban area while 3 (4.1%) stay in rural areas.

#### Instruments for data collection

A questionnaire and an interview guide were used for data collection. The instruments were constructed by the author based on the conceptual and theoretical considerations of the paper.

##### Description of questionnaire

The questionnaire was constructed to find out information about resilient cultural practices and cognitive development. It was made of three sections.

Section one is composed of demographic questions with respect to the age, gender, and place of residence of respondents.

Section two composed of variables and items in relation to resilient cultural practices (Socio-emotional values = 7 items; Socio-moral values = 10 items; Personal survival assets = 7 items; Responsibility/Leadership = 8 items).

Section three composed of variables and items in relation to cognitive development (cognitive processes = 5 items; cognitive reasoning = 4 items; cognitive skills = 5 items; cognitive strategy = 3 items).

The items in sections two and three were rated on a Likert scale of 5 score points based on the occurrence of practices and behaviours as follows:

Always = 5 points.

Frequently = 4 points.

Sometimes = 3 points.

Scarcely = 2 points.

Never = 1 point (see Appendix 1 for the complete questionnaire).

##### Description of interview guide

The interview guide was constructed to find out information on learning pathways that are relevant to children in African cultures. In this regard, a question was asked to respondents in relation to the identification of physical learning pathways, social learning pathways, and psychological learning pathways. A further question to respondents to explain why they view them as enhancing dimensions for cognitive development through cultural practices that are resilient focused. During the interview process, further probing questions were asked to respondents to solicit more information on the learning pathways. Note-taking and tape recorders were used to collect the qualitative data after which they were analysed through the method of thematic content analysis (See Appendix 2 for the complete interview guide).

#### Reliability of questionnaire

The reliability statistics showed that the internal consistency of respondents was satisfactory for all variables with 0.818 obtained for cognitive development and 0.867 for resilient cultural practices. The overall reliability of the questionnaire is 0.867 above the recommended threshold of 0.7. Therefore, the questionnaire and all its items were reliable for the study (Table 2).

**Table 2:** Reliability of questionnaire.

Main variable	Sub-variables	Cronbach Alpha coefficient	Variance	Items
Cognitive development	Cognitive processes	0.865	0.009	5
	Cognitive reasoning	0.747	0.015	4
	Cognitive skills	0.769	0.128	5
	Cognitive strategy	0.799	0.023	3
	**Sub-overall**	**0.818**	**0.047**	**17**
Resilient cultural practices	Socio-Emotional	0.839	0.143	7
	Socio-moral	0.923	0.007	10
	Personal survival assets	0.804	0.025	7
	Responsibility/leadership skills	0.865	0.031	8
	**Sub-overall**	**0.867**	**0.035**	**32**
**Overall reliability coefficient**		**0.876**	**0.054**	**62**

#### Method of analysis

##### Analysis of quantitative data

As for the quantitative data, a pre-designed EpiData Version 3.1 (EpiData Association, Odense Denmark, 2008) database which had in-built consistency and validation checks was used to enter the data. Further consistency, data range, and validation checks were also performed in SPSS version 21.0 (IBM Inc., 2012) to identify invalid codes. Data were analysed using frequency and proportions and Multiple Regression Analysis to aggregate scores for given conceptual components.

##### Analysis of qualitative data

The analysis of qualitative data was done following the systematic process of thematic and content analysis (Weber, 1990). The first stage involved planning on the level of analysis. At this level, single words, clauses, and sets of words or phrases were coded. A decision was made on how many different concepts to code. This involved developing pre-defined or interactive sets of concept categories. A code list earlier developed based on the major indicators of the study was made. Relevant categories not included in the initial code list were added during the coding process (*in vivo* coding). Introducing this coding flexibility allowed for new, important material to be incorporated into the coding process that could have significant bearings on results. During the coding, it was assumed that any idea that emerges at least once is relevant. The analysis is presented in a thematic table.

### Findings

The findings of the study are presented in three dimensions. These embody the presentation of multiple responses set of the independent variable (resilient cultural practices) and dependent variable (cognitive development) of the study. This is followed by the display of multiple regression analysis on the effects of resilient cultural practices on various components of cognitive development. Finally, there is the presentation of thematic content analysis on learning pathways that are relevant to children in African cultures.

#### Multiple responses set of the independent and dependent variables of the study.

**Figure 3:**
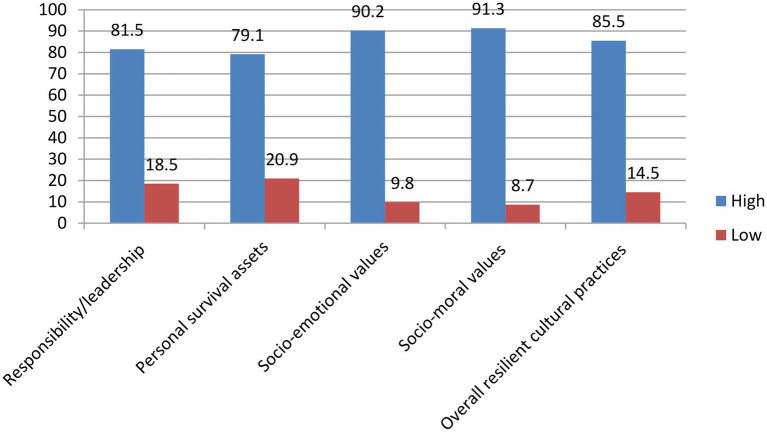
Respondents’ appraisal of resilient cultural practices.

Figure 3 shows that 85.5% of respondents are high in their resilient cultural practices whereas 14.5% of them are low in resilient cultural practices.

**Figure 4:**
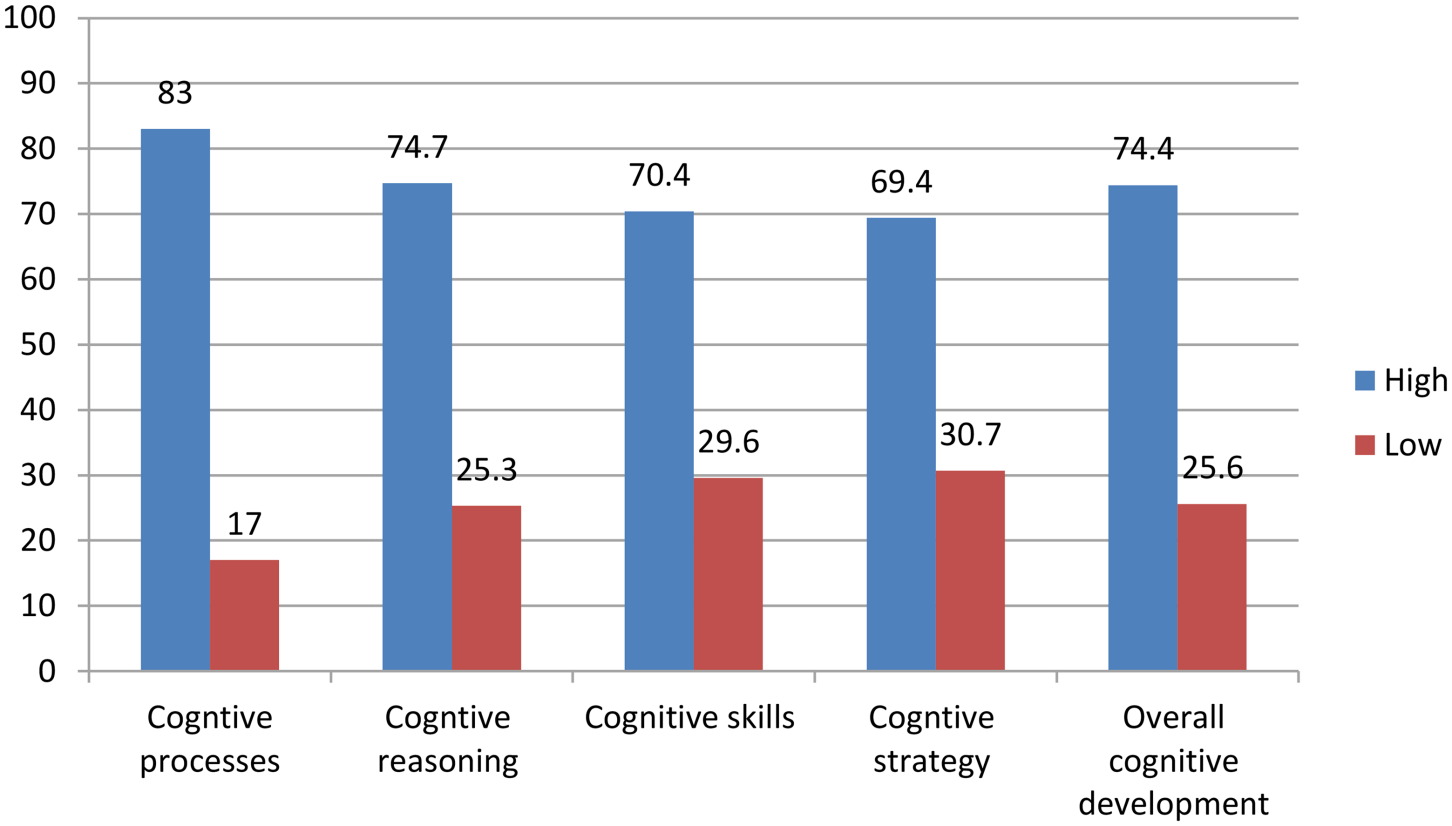
Respondents’ appraisal of cognitive development.

Overall, statistics in Figure 4 show that 74.4% of respondents’ cognitive development is high while a proportion of them 25.6% are low.

#### Multiple regression analysis on the effects of resilient cultural practices on various components of cognitive development

##### Resilient cultural practices and cognitive processes

The results of the regression in Table 3 indicated that the model explained.499 (49.9%) of the variance and that the model was a significant predictor of cognitive process, F (16.947, *p* = 0.000). Specifically, the regression coefficients indicated that: (a) Socio-emotional values contributed significantly to the model (B = 0.163, *p* = 0.000), meaning that a unit increase in socio-emotional values led to a 0.163 increase in cognitive processes; (b) Socio-moral values contributed significantly to the model (B = 0.104, *p* = 0.000), meaning that a unit increase in socio-moral values let to a 0.104 increase in cognitive processes; (c) personal survival assets contributed significantly to the model (B = 0.165, *p* = 0.000), meaning that a unit increase in personal survival assets led to a 0.165 increase in cognitive processes; (d) Responsibility/leadership contributed significantly to the model (B = 0.105, *p* = 0.000), meaning that a unit increase in responsibility/leadership led to a.105 increase in cognitive processes.

**Table 3:** Regression analysis estimating the effect of resilient cultural practices on cognitive processes.

	Unstandardized Coefficients		Standardized Coefficients	***t*** test	value of ***p***
	B	Std. Error	Beta		
(Constant)	−5.806	0.771		−7.535	0.000
Socio-emotional	0.163	0.017	0.616	9.389	0.000
Socio-moral	0.104	0.013	0.567	8.251	0.000
Personal survival assets	0.165	0.015	0.671	10.862	0.000
Responsibility/leadership	0.105	0.014	0.529	7.479	0.000
**Model summary**					
R	0.707				
R-square	0.499				
Adjusted R-square	0.470				
Std. Error of the Estimate	0.72817054				
**ANOVA**					
***F*-value**			16.947		
**value of** ***p***			0.000		

Dependent variable: Cognitive processes.

##### Resilient cultural practices and cognitive reasoning

Table 4 indicates that the model explained.407 (40.7%) of the variance and that the model was a significant predictor of cognitive reasoning, F (11.664, *p* = 0.000). Specifically, the regression coefficients indicated that: (a) Socio-emotional values contributed significantly to the model (B = 0.105, *p* = 0.000), meaning that a unit increase in socio-emotional values led to a 0.105 increase in cognitive reasoning; (b) Socio-moral values contributed significantly to the model (B = 0.088, *p* = 0.001), meaning that a unit increase in socio-moral values led to a 0.008 increase in cognitive reasoning; (c) personal survival assets contributed significantly to the model (B = 0.631, *p* = 0.000), meaning that a unit increase in personal survival assets led to a 0.631 increase in cognitive reasoning; (d) Responsibility/leadership contributed significantly to the model (B = 0.488, *p* = 0.000), meaning that a unit increase in responsibility/leadership led to a.488 increase in cognitive reasoning.

**Table 4:** Regression analysis estimating the effect of resilient cultural practices on cognitive reasoning.

	Unstandardized Coefficients		Standardized Coefficients	t-test	value of p
	B	Std. Error	Beta		
(Constant)	−4.416	0.839		−5.266	0.000
Socio-emotional	0.105	0.029	0.397	3.643	0.001
Socio-moral	0.088	0.019	0.482	4.633	0.000
Personal survival assets	0.631	0.092	0.631	6.847	0.000
Responsibility: Leadership	0.488	0.104	0.488	4.706	0.000
**Model Summary**					
R	0.638				
R-square	0.407				
Adjusted R-square	0.372				
Std. Error of the Estimate	0.79244937				
**ANOVA**					
**F-value**	11.664				
***value of p***	0.000				

Dependent Variable: Cognitive reasoning.

##### Resilient cultural practices and cognitive skills

The findings in Table 5 indicates that the model explained.354 (35.4%) of the variance and that the model was a significant predictor of cognitive skills, F (9.335, *p* = 0.000). Specifically, the regression coefficients indicated that: (a) Socio-emotional values contributed significantly to the model (B = 0.119, *p* = 0.000), meaning that a unit increase in socio-emotional values led to a.119 increase in cognitive skills; (b) Socio-moral values contributed significantly to the model (B = 0.076, *p* = 0.000), meaning that a unit increase in socio-moral values led to a 0.076 increase in cognitive skills; (c) personal survival assets contributed significantly to the model (B = 0.133, *p* = 0.000), meaning that a unit increase in personal survival assets led to a 0.133 increase in cognitive skills; (d) Responsibility/leadership contributed significantly to the model (B = 0.100, *p* = 0.000), meaning that a unit increase in responsibility/leadership led to a.100 increase in cognitive skills.

**Table 5:** Regression analysis estimating the effect of resilient cultural practices on cognitive skills.

	Unstandardized Coefficients		Standardized Coefficients	***t*** test	value of ***p***
	B	Std. Error	Beta		
(Constant)	−4.377	0.875		−5.003	0.000
Socio-emotional	0.119	0.028	0.448	4.227	0.000
Socio-moral	0.076	0.020	0.417	3.869	0.000
Personal survival assets	0.133	0.025	0.541	5.423	0.000
Responsibility: Leadership	0.100	0.020	0.503	4.899	0.000
**Model summary**					
R	0.595				
R-square	0.354				
Adjusted R-square	0.317				
Std. Error of the Estimate	0.82674026				
**ANOVA**					
***F*-value**	9.335				
**value of** ***p***	0.000				

Dependent Variable: Cognitive skills.

##### Resilient cultural practices and cognitive strategy

The findings in Table 6 indicate that the model explained.559 (55.9%) of the variance and that the model was a significant predictor of cognitive strategy, F (21.578, *p* = 0.000). Specifically, the regression coefficients indicated that: (a) Socio-emotional values contributed significantly to the model (B = 0.142, *p* = 0.000), meaning that a unit increase in socio-emotional values led to a 0.142 increase in cognitive strategy; (b) Socio-moral values contributed significantly to the model (B = 0.119, *p* = 0.000), meaning that a unit increase in socio-moral values led to a 0.119 increase in cognitive strategy; (c) personal survival assets contributed significantly to the model (B = 0.173, *p* = 0.000), meaning that a unit increase in personal survival assets led to a 0.173 increase in cognitive strategy; (d) Responsibility/leadership contributed significantly to the model (B = 0.135, *p* = 0.000), meaning that a unit increase in responsibility/leadership led to a.135 increase in cognitive strategy.

**Table 6:** Regression analysis estimating the effect of resilient cultural practices on cognitive strategy.

	Unstandardized Coefficients		Standardized Coefficients	***t*** test	value of ***p***
	B	Std. Error	Beta		
(Constant)	−5.824	0.723		−8.058	0.000
Socio-emotional	0.142	0.027	0.537	5.369	0.000
Socio-moral	0.119	0.017	0.651	7.222	0.000
Personal survival assets	0.173	0.021	0.700	8.269	0.000
Responsibility: Leadership	0.135	0.017	0.680	7.806	0.000
**Model summary**					
R	0.748				
R-square	0.559				
Adjusted R-square	0.533				
Std. Error of the Estimate	0.68307187				
**ANOVA**					
***F*-value**	21.578				
**value of** ***p***	0.000				

Dependent variable: Cognitive strategy.

##### Overall resilient cultural practices and cognitive development

The overall findings in Table 7 indicate that the model explained.559 (55.9%) of the variance and that the model was a significant predictor of overall cognitive development, F (25.399, *p* = 0.000). Specifically, the regression coefficients indicated that: (a) Socio-emotional values contributed significantly to the model (B = 0.1.548, *p* = 0.000), meaning that a unit increase in socio-emotional values led to a 1.548 increase in overall cognitive development; (b) Socio-moral values contributed significantly to the model (B = 1.109, *p* = 0.000), meaning that a unit increase in socio-moral values led to a 1.109 increase in overall cognitive development; (c) personal survival assets contributed significantly to the model (B = 1.798, *p* = 0.000), meaning that a unit increase in personal survival assets led to a 1.798 increase in overall cognitive development; (d) Responsibility/leadership contributed significantly to the model (B = 1.247, *p* = 0.000), meaning that a unit increase in responsibility/leadership led to a 1.247 increase in overall cognitive development.

**Table 7:** Regression analysis estimating the effect of resilient cultural practices on overall cognitive development.

	Unstandardized Coefficients		Standardized Coefficients	***t*** test	value of ***p***
	B	Std. Error	Beta		
(Constant)	10.056	6.701		1.501	0.138
Socio-emotional	1.548	0.245	0.601	6.333	0.000
Socio-moral	1.109	0.165	0.623	6.704	0.000
Personal survival assets	1.798	0.188	0.751	9.584	0.000
Responsibility: Leadership	1.247	0.175	0.646	7.135	0.000
**Model summary**					
R	0.774				
R-square	0.599				
Adjusted R-square	0.575				
Std. Error of the Estimate	6.33438				
**ANOVA**					
***F*-value**	25.399				
**value of** ***p***	0.000				

Dependent variable: Overall cognitive development.

A summary of the results from multiple regression analysis of the four main constructs of cognitive development for this study, demonstrated that resilient cultural practices impact more cognitive strategy (55.9%) followed by process (49.9%), then reasoning (40.7%), and cognitive skills (34.4%) the lowest. Cognitive strategy and process are the constructs most affected by resilience cultural practices.

#### Thematic content analysis on learning pathways that are relevant for cognitive enrichment of children in African cultures

***Question one: Please identify at least three such activities that you engage children in each pathway specified below.***

- ***Physical learning pathways.***
- ***Social learning pathways.***
- ***Psychological learning pathways.***

Table 8 above identifies the physical, social, and psychological learning that are relevant to children in African cultures. The emerging themes include the availability of an enabling rich interactive environment; Participation in physical activities and building objects; Engaging children in daily chores; Monitoring, feedback, and reinforcement; direct teaching; Engaging children in storytelling, games, play, songs, and reenacting children through interest-driven activities, prior knowledge or experiences and learning opportunities.

**Table 8:** Learning pathways.

**Category**	**Themes**	**Sample quotation**
Physical learning pathways	Enabling a rich interactive environment	*Creating an enabling rich interactive environment with resources that would tap and stimulate the cognitive resources available to the child. Verbal, nonverbal, and material resources must be relevant and meaningful, and above all stimulating.*
	Physical activities	*taking care of younger siblings and engaging in play and dance*
	Building objects	*Building home and toy objects with mud, sticks, and stones*
	Engaging children in daily chores	*Engaging children in cooking, farming, selling, and cleaning the home and environment.*
Social learning pathways	Monitoring, feedback, reinforcement	*Through monitoring, feedback, reinforcement, and gradually changing tasks to be performed from simple to more complex*
	Teaching	*By teaching them the cultural practices that reflect our character and value, especially in respecting one another like the elderly ones, greetings, and helping those in need. Secondly, increase the well-being of the children by being a good example to them in the way I do things*
	Storytelling	*Parents and children will seat down in the evening and tell stories about their culture. These activities equally entail telling the meaning of proverbs and riddles*
	Games and play songs	*Children engage in cultural games and play songs where they learn a lot of cognitive and interpersonal skills*
Psychological learning pathways	Interest driven	*This is because most of what they do they learn by participating. Most African parents involve their children in diverse activities at an early stage. As they grow, they continue to carry out these activities which make them become independent.*
	Prior knowledge or experiences	*The African child is raised at a very tender age to be an adult, so they engage in situational and contextual activities which give them prior knowledge or experiences on which to base their learning even in later life.*
	Learning opportunities	*The fact that they are often given the opportunity to engage in the task as well as participate with more knowledgeable others, makes them develop the skills to direct their own learning.*

***Question two: Explain why you view the learning pathways as enhancing dimensions for cognitive development through cultural practices that are resilient-focused.***

Box 1: Sample Responses.

*R1: The cultural values of interdependence provide opportunities where social, physical, and psychological actions interact and interplay to ensure the use of cognitive skills of creativity, imagination, and critical and analytical. It is built on the holistic view of development. Cognitive development cannot be isolated though informed by the individual’s mind theory no doubt, but also by actions and activities engaging all of the above pathways.*

*R2: While engaging in the task, they develop physical, social, and psychological skills that are cognitively oriented. This is seen in the progressive manner they understand and perform these tasks.*

*R3: When children engage in those activities, especially as they interact with each other, it enhances language development, divergent thinking, imagination, problem-solving,* etc.

*R4: They help the child to grow healthy and responsible in society. It gives the children a holistic informal education that helps them in future pursuits.*

*R5: Prayers, access to high dimensions of cognitive development from the supernatural. Taking children to farm together with parents instills a culture of hard work.*

*R6: They learn easily, through this, they develop and build language skills and increase vocabulary, learn to memorise and store some information for easy retrieval if and when need be, for usage.*

*R7: Social activities develop social skills necessary for cooperative work. They develop competencies that make them responsive as independent learners and as members of a team. Children naturally like doing things in groups. They face challenging activities as a group with individuals making suggestions to solve the problem. They take turns without knowing. They hardly give up and tend to be very resilient.*

*R8: Playing promote a child’s brain development by providing crucial life experiences to set the grounds for brain growth and it equally gives the child confidence to keep exploring and learning about the world.*

*R9: Exercising can help boost thinking and memory indirectly by improving one’s mood and reducing stress, depression, and anxiety.*

*R10: During play, they interact with each other, and this improves their communicative skills, and problem-solving skills as they remain resilient and focused and try to avoid errors. The weak ones imitate the smart ones, and this enhances their cognitive development.*

*R11: House chores help children develop self-confidence, time consciousness, and independence as they skillfully work on tasks assigned to them. Certain tasks must be carried out at a given time. This obviously develops them cognitively and keeps them resilient.*

### Limitations of the study

The following limitations were encountered during the study

- Due to the outbreak of the COVID-19 pandemic, the questionnaire was strictly answered online. This brought a limitation in the sample size for the study.
- The sample was equally made of mostly urban settlers since they had access to the internet more than rural settlers.

## Discussion, conclusion, and recommendation

The findings of this research substantiated the fact that children and learners actively participate collaboratively in actions and activities enhancing their cognitive development. African cultural values implicitly or explicitly inform socialisation activities that enable children to be involved in all developmental tasks in the family, school, and neighbourhood. Resilient cultural practices have a relationship with Africentric learning pathways and these equally have a positive effect on the cognitive development of children. These fall in line with earlier studies carried out by Yatta (2007) when he found that leadership ceremonial practices enhanced children’s reasoning. Similarly, Sternberg et al. (2001); Yatta (2007), equally found out that the development of cognitive skills and processes was enhanced by resilient cultural practices of sense of responsibility and personal survival skills. Through socio-emotional and moral practices like indigenous games and play and songs, children were able to develop cognitive strategies in interpersonal relations (Nyota and Mapara, 2008).

Hence, resilient cultural practices constitute the basis for equipping children with skills and competencies that enrich and strengthen their cognitive functioning and allow for flexibility to be able to initiate, create, invent, and do all that only children exposed to such enriched psychological development can perform. So, in the Africentric resilient cultural practices, strategies and tasks are used by both parents, teachers, and children through actions such as giving and taking; and negotiating for behavioural change. Most of the respondents indicated that the use of African resilient practices promotes personal survival assets, non-verbal communication skills, and assuming responsibility/leadership when necessary. The research supports that there is a strong relationship between Africentric values, resilient cultural practices, and learning pathways. The qualitative findings illustrated that African children manipulate their physical, social, and psychological pathways. The seamless hypothetical pathways model emerges as an outcome of this study. There is a relationship between learning pathways and cognitive development/dimensions. The findings confirmed the hypothesis that the invisible, yet powerful learning pathways through the diverse activities with symbols, tools, and interpersonal interactions develop children’s cognitive reasoning, skills, functioning, and strategies.

Views on Learning Pathways for Cognitive Development are in the epistemology of how Africans perceived the education and training of their children. On this account, the cultural environment whereby the pathways exist provides authentic relevant conditions for children to participate. When children participate in their daily chores, and in all cultural amplifiers, at home, school playground, neigbourhood, farm, and market, they pass through the physical, social, and psychological pathways, providing solutions to problems, constructing new insights, new relationships, and representations, and a better understanding of their cultural context. Mediated mutual reciprocal theory encourages children’s active contribution through mutual reciprocated experiences as they journey through the rich learning pathways.

Cognitive growth involves an interaction between basic human capabilities and different ecologies, culturally invented technologies, and activities that serve as amplifiers of these capabilities. From the Africentric resilient cultural practices, it is evident that knowing and reasoning must emerge from the children’s individual appropriation of their cognitive and cultural resources for meaningful learning and understanding (Cole, 1990). A significant contribution of this research is the knowledge that children/learners contribute actively through quality involvement in different actions through learning pathways for their cognitive development. Conclusively the cultural learning pathways model postulates that children maximally employ their cognitive processes without adult knowledge that contributes to their cognitive development. Harnessing local knowledge is important in prioritising the local community as the object of cognitive development in children (Gauvain et al. (2011)). Resilient Cultural practices in the socialisation of the African child ensure the development of responsible, leadership, and personal survival skills directed by African values and beliefs. These values and beliefs set out to encourage and promote socio-emotional skills to enable children to have control over their emotions through empathy. They also addressed socio-moral skills to ensure the development of the spirit of the harmonious communal culture valuable for good citizenship development. The enhanced cognitive development focus on skills such as reasoning, thinking, reflecting, creativity, and imagination and using strategies such as communication, collaboration, and non-verbal communication. Clearly, developing cognitive strategy and focusing on processes seem to be of great importance in development and learning. This paper has provided some insights into the psychology of child development as a whole and specifically cognitive development from an Africentric perspective. This paper equally, ascertains those resilient practices built on African cultural values to promote cognitive development during childhood within learning pathways using the Mediated Mutual Reciprocity as an explanatory theory. Finally, the link between pathways, resilient cultural practices, and cognitive development highlights the significance of children’s involvement through participation.

## Data availability statement

The original contributions presented in the study are included in the article/Supplementary material,; further inquiries can be directed to the corresponding author.

## Author contributions

The author confirms being the sole contributor of this work and has approved it for publication.

## Conflict of interests

The authors declare that the research was conducted in the absence of any commercial or financial relationships that could be construed as a potential conflict of interest.

## Publisher’s note

All claims expressed in this article are solely those of the authors and do not necessarily represent those of their affiliated organizations, or those of the publisher, the editors and the reviewers. Any product that may be evaluated in this article, or claim that may be made by its manufacturer, is not guaranteed or endorsed by the publisher.

## Footnotes

^1^Noumsi, N. (2021). Sculpture Designer, of the Pictures in the text, Fondation Noumsi Yaounde, (Unpublished).
