# Supplementary material for: Resilient cultural practices for cognitive development during childhood within learning pathways with support from mediated mutual reciprocal theory
Source: Front Psychol. 2023 Mar 27;13:994156. doi: 10.3389/fpsyg.2022.994156 (PMC10084727; doi:10.3389/fpsyg.2022.994156)
Supplement: Supplementary file 1 [file Presentation_1.pdf]

APPENDIX 1: QUESTIONNAIRE

This Questionnaire has three main parts, the first deals with demographic information, the second with resilient cultural practices and the third on cognitive development. Please read each statement and place a tick (√) on the appropriate box of your choice on a number starting with the highest 5, 4, 3, 2 and 1 the lowest indicating your weighting of the statements. There are no correct or wrong answers. You are guaranteed of confidentiality.

The rating scale is as follows:

5 Always
4 Frequently
3 Sometimes
2 Scarcely
1 Never

A. Demographic Information

Age: ______________________

Gender: Female ☐ M ☐

Location: Rural ☐ Urban ☐

B. Resilient cultural practices

SN		5 Al	4 Fr	3 So	2 Sc	1 Nv
	**Socio-Emotional values**					
1	Children should see challenges as opportunities to demonstrate abilities to learn to share the little they have with those who do not have in times of want.					
2	Elders must be respected by children and must not be addressed by their names.		.			
3	Children should stand up and give their seats for the elderly and the weak or sick					
4	Children should take care of properties whether they are theirs or those of others					
5	Children must love one another as it brings joy and Happiness					
6	Children must master their emotion by telling them to avoid shouting at others when things go wrong or when they are hurt.					
7	Children should accept others even those that are different from them such the disabled or the poor					

	**Socio-moral values**					
1	Children must have compassion for others who have problems for example failed exams.					
2	Children must build the communal spirit of living together in peace and harmony to ensure happiness					
3	Children must interact positively with siblings and peers					
	and family members.					
4	Children should value reconciliation whenever there is problem especially when they are hurt or when someone hurts them					
5	Children must value giving others a sense of belonging within the family setting and eventually in school in particularly checking on them.					
6	Children must demonstrate moral issues such as honesty and uprightness so they can be trusted.					
7	Children must manifest good behaviour always with a sense of dignity to gain self-respect.					
8	Children must know the importance of obeying rules, if not, they will get into trouble.					
9	Children must have sense of social justice, so they learn not to inflict pain on others by depriving them of their rights.					
10	Children should know how to appreciate					
	**Personal Survival Assets**					
1	I insist that children learn to complete any tasks they start such as pilling up washing of their dresses or homework.					
2	I train children on the importance of being responsible by getting up on time, taking care of younger siblings and do their schoolwork well and on time					
3	I educate children to learn to control their emotions by avoiding quarreling and fighting in school and home even neighbourhood.					
4	I train children to develop the ability to observe problems and learn to provide solutions.					
5	I teach children that hard work is very important by giving them tasks to execute					
6	I stimulate children’s mind by telling them cultural stories, folklores, and proverbs to teach them the underlying morals.					
7	I help my children to learn to analyze their perceptions of any situation when making certain decisions or taking a position.					
	**Responsibility/Leadership**					
1	I teach children the habit of appreciating cleanliness, by making them clean the compound and the house.					
2	I insist on children making their beds and tidy their personal belongings					

3	I make children to plan and prepare family meals so that in					
	my absence or other adults can take care of the home					
4	I send children on errands to the market to buy certain things, to enable develop bargaining skills.					
5	I educate children to take care of younger siblings by bathing, feeding, playing with them, and putting them to bed					
6	I encourage children to co-create leadership skills in family matters by taking initiative at home and school and setting family rules					
7	I insist on the value of manual work on gardens and farms including the socio-economic activities					
8	I help build their self-confidence by encouraging them to face challenges such as not having regular food and not being able to afford all required textbooks					

C. Cognitive Development

SN	Items<br>Cognitive development	5	4	3	2	1
		Al	Fr	So	Sc	Ne
	**Cognitive processes**					
1	Help children to simplify complex issues into smaller parts for better understanding through working with them on specific meaningful task					
2	Develop children's ability to combine different issues, ideas, and things as dance, music, drum characterising knowledge about cultural festivals		.			
3	Help children solve problems by using valuable information such as discussing buying, selling and getting change or making profit and loss in selling					
4	Educate children to work towards accomplishing desired goals such as in doing homework, the task must be completed well on time.					
5	Help my children to learn to analyze their own thoughts process in decisions making.					
	**Cognitive reasoning**					
1	Train children to learn to understand a given task, organize, plan, and priorities before engagements in solving it					
2	Educate children to analyze situation before they act such as thinking before answering questions or sharing information					
3	Encourage children to think about consequences before staying away from school or home.					
4	Ask them to interpret the values obtained from experiences in doing home and school activities for their development					

	**Cognitive skills**					
1	Train children on how to retain and mentally manipulate information by trying to first understand then memories					
2	Always ask children to explain what happened when they went to the farm to encourage experiences sharing through communication.					

3	Encourage children to always pay attention by listening keenly and being observant.					
4	Educate children to learn to ask thoughtful questions such as why do we have to pray every day?					
5	Teach my children to see every situation and tasks as a learning situation as helping in selling in the market or cleaning the compound					
	**Cognitive strategy**					
1	Encourage children to obtain mastery through practices in working in the farm, learning how to hoe, weed, plant and harvest.					
2	Teach children the value of participating with others to learn through group work in all learning environment.					
3	Hold meaningful, truthful conversations with my children to enable them learn quality interactive skills					

APPENDIX 2: INTERVIEW GUIDE

1) Please identify at least three such activities that you engage children in each pathway specified below
   a) Physical learning pathways
   b) Social learning pathways
   c) Psychological learning pathways
2) Explain why you view them as enhancing dimensions for cognitive development through cultural practices that are resilient-focused.
